# Physiology and Pathophysiology of Compensatory Adaptations of a Solitary Functioning Kidney

**DOI:** 10.3389/fphys.2020.00725

**Published:** 2020-06-26

**Authors:** Zoe McArdle, Michiel F. Schreuder, Karen M. Moritz, Kate M. Denton, Reetu R. Singh

**Affiliations:** ^1^Cardiovascular Program, Monash Biomedicine Discovery Institute and Department of Physiology, Monash University, Melbourne, VIC, Australia; ^2^Department of Pediatric Nephrology, Amalia Children’s Hospital, Radboud University Medical Center, Nijmegen, Netherlands; ^3^Child Health Research Centre and School of Biomedical Sciences, University of Queensland, Brisbane, QLD, Australia

**Keywords:** solitary functioning kidney, compensatory hypertrophy, glomerular hyperfiltration, renal sympathetic nerves, renin angiotensin system, nitric oxide

## Abstract

Children born with a solitary functioning kidney (SFK) have an increased risk of hypertension and kidney disease from early in adulthood. In response to a reduction in kidney mass, the remaining kidney undergoes compensatory kidney growth. This is associated with both an increase in size of the kidney tubules and the glomeruli and an increase in single nephron glomerular filtration rate (SNGFR). The compensatory hypertrophy and increase in filtration at the level of the individual nephron results in normalization of total glomerular filtration rate (GFR). However, over time these same compensatory mechanisms may contribute to kidney injury and hypertension. Indeed, approximately 50% of children born with a SFK develop hypertension by the age of 18 and 20–40% require dialysis by the age of 30. The mechanisms that result in kidney injury are only partly understood, and early biomarkers that distinguish those at an elevated risk of kidney injury are needed. This review will outline the compensatory adaptations to a SFK, and outline how these adaptations may contribute to kidney injury and hypertension later in life. These will be based largely on the mechanisms we have identified from our studies in an ovine model of SFK, that implicate the renal nitric oxide system, the renin angiotensin system and the renal nerves to kidney disease and hypertension associated with SFK. This discussion will also evaluate current, and speculate on next generation, prognostic factors that may predict those children at a higher risk of future kidney disease and hypertension.

## Introduction

Congenital anomalies of the kidney and urinary tract (CAKUT) represent the primary cause of chronic kidney disease (CKD) in the pediatric population, accounting for approximately 50% of cases (Becherucci et al., 2016). A solitary functioning kidney (SFK) is a common abnormality in the spectrum of CAKUT and is characterized by a reduction in kidney mass and nephron number. For children born with a SFK, or who undergo unilateral nephrectomy early in life, the risk of hypertension, kidney injury and CKD is increased from early in life. As such, approximately 50% of children born with a SFK develop hypertension as early as the age of 18 years and 20–40% require dialysis by the age of 30 years (Sanna-Cherchi et al., 2009; Westland et al., 2013a). In contrast, in long-term studies it has been demonstrated that kidney function is well preserved following donation of a kidney in adulthood (Kasiske et al., 1995; Fehrman-Ekholm et al., 2001; Ibrahim et al., 2009). However, recent studies have reported that kidney donation can result in a small increase in the risk of kidney failure compared with healthy non-donors (Muzaale et al., 2014). Additionally, an increase in risk of kidney disease after donation is also observed in kidneys donors with pre-existing conditions such as obesity (Niemi and Mandelbrot, 2014). The mechanisms that underlie the differences in prognosis for loss of a kidney early in life compared with adulthood are not well understood but may be associated with differences in compensatory adaptations to loss of kidney mass. Following a reduction in kidney mass, the remaining kidney undergoes adaptive compensatory hypertrophy, of both the tubules and the glomeruli, and compensatory glomerular hyperfiltration to maintain kidney function. However, the mechanisms that facilitate these compensatory adaptations following a reduction in kidney mass may in the long-term contribute to the onset of hypertension and kidney injury. Although common prognostic indicators of kidney injury [hypertension, proteinuria, estimated GFR (eGFR) < 60 ml/min/1.73 m^2^] have been observed in children born with an SFK from as early as 10 years of age, early markers at time of diagnosis of SFK, that distinguish those at an elevated risk of kidney injury are needed (Schreuder et al., 2008; Westland et al., 2011, 2013a).

## Prevalence of a SFK

Unilateral renal agenesis (URA) and multicystic dysplastic kidney (MCDK) are the two main abnormalities in the spectrum of CAKUT that result in a SFK (Schreuder et al., 2009; Westland et al., 2013b). There is a higher incidence of CAKUT in males compared with females (Kummer et al., 2012; Tain et al., 2016; Li et al., 2019). In a Taiwanese study, males had a 1.83 greater risk of CAKUT compared with females (Tain et al., 2016). Additionally, males with CAKUT advance to renal replacement therapy earlier than their female counterparts (Wuhl et al., 2013). URA is estimated to occur in 1 in ∼2000 births (Westland et al., 2013b). MCDK is a severe form of renal dysplasia that can result in a non-functioning kidney and has an estimated incidence of 1 in ∼4300 births (Schreuder et al., 2009). Both URA and MCDK are conditions of SFK that are congenital in origin. However, a SFK can also be acquired early in life following unilateral nephrectomy, which can be due to oncological (e.g., Wilm’s tumor) or non-oncological causes (e.g., CAKUT such as pelviureteric junction obstruction, posterior urethral valves or vesicoureteral reflux) (Westland et al., 2013a; Mavinkurve-Groothuis et al., 2016).

## Clinical Outcomes

The clinical outcomes of children with a congenital SFK or early-acquired SFK remain contentious. Data on the long-term cardiovascular and renal consequences of a congenital SFK are limited (summarized in Table 1) (Argueso et al., 1992; Sanna-Cherchi et al., 2009; Wang et al., 2010; Xu et al., 2019). In a longitudinal study it was observed that of 71 patients with a congenital SFK ∼20–40% had begun dialysis by the age of 30 years (Sanna-Cherchi et al., 2009). In a Chinese study of 48 adults with a congenital SFK, 38.5% had reduced GFR (<60 ml/min/1.73 m^2^), 35.4% had proteinuria and two individuals had begun dialysis by a mean age of ∼37 years (Wang et al., 2010). Similarly, Xu et al. (2019) found that of 118 patients with URA, 43% had proteinuria and 25% had a reduced GFR (<60 ml/min/1.73 m^2^) at a median age of 32 years. These studies indicate that there is a progressive loss of kidney function in individuals with a congenital SFK throughout adulthood, and in some individuals results in progression to kidney failure by 30–40 years of age.

**TABLE 1:** Summary of clinical outcomes in patients with a congenital or early-acquired SFK.

References	Total number of patients	Gender (M/F)	Type of patients included	Age (years)	Kidney injury	Kidney length as a predictor of kidney injury
Sanna-Cherchi et al. (2009)	71	52/19	URA or empty renal fossa upon imaging	Mean age 21 (SD 13.6)	20–40% dialysis	NR
Wang et al. (2010)	65	36/29	48 with URA 17 with severe unilateral kidney dysplasia	Mean age 36.7 (SD 13.1)	38.5% reduced GFR (<60 ml/min/1.73 m^2^) 35.4% proteinuria 3% dialysis 36.9% hypertension	Kidney length < 120 mm five-fold greater risk reduced GFR
Xu et al. (2019)	118	62/56	URA	Median age 32	25.4% reduced GFR (<60 ml/min/1.73 m^2^) 43% proteinuria 32.2% hypertension	Patients with reduced GFR had a shorter kidney length
Westland et al. (2011, 2013a)	407	265/142	223 congenital SFK (URA or MDCK) 184 early acquired SFK	Mean age 6.4 (SD 5.7) Mean age 9.8 (SD 5.6) Mean age 4.9 (SD 5.4)	6% reduced GFR (<60 ml/min/1.73 m^2^) 19% proteinuria 26% hypertension	Patients with a smaller SFK had a greater incidence of kidney injury
Marzuillo et al. (2017, 2019)	306	Not specified	Prenatally diagnosed (URA and MCDK)	Mean age of kidney injury onset 8.2 (SD 5.6)	1.3% reduced GFR (<90 ml/min/1.73 m^2^) 3.6% proteinuria 0.6% hypertension	In 162 of the 306 patients - kidney length < 2-SDS in the neonate period had a greater risk of reduced GFR at 15 years of age irrespective of postnatal kidney hypertrophy
La Scola et al. (2016)	146	95/51	URA, aplasia and MCDK	Median age 2.2 Mean age 5.6 ± (4.4 SD) Mean age 12.2 (3.1 SD)	12% reduced GFR (<90 ml/min/1.73 m^2^) 4% proteinuria 5% hypertension	Kidney length < 10% expected for a congenital SFK was the strongest predictor of reduced GFR by 10 years of age

*NR, not recorded; SD, standard deviation; URA, unilateral renal agenesis; MCDK, multicystic dysplastic kidney; GFR, glomerular filtration rate; eGFR, estimated glomerular filtration rate.*

The Kidney of MONofunctional Origin (KIMONO) longitudinal study is currently the largest cohort, with over 400 children with both congenital and early-acquired SFK, based in The Netherlands (Schreuder et al., 2008; Westland et al., 2011, 2013a). It was designed to study the prognosis of a SFK of different origins in childhood (Schreuder et al., 2008; Westland et al., 2011, 2013a). The KIMONO cohort comprised of patients with a ‘primary’ SFK of congenital origin, including both URA and MCDK, and a ‘secondary’ SFK of early-acquired origin following unilateral nephrectomy due to obstructive or reflux nephropathy (Westland et al., 2011, 2013a). These groups were further subdivided into those with and without ipsilateral CAKUT (in the remnant kidney) (Westland et al., 2011, 2013a). In this study kidney injury was defined as hypertension, proteinuria, reduced eGFR and/or the use of kidney protective medication [e.g., Angiotensin converting enzyme inhibitor (ACEi) or Angiotensin II receptor blockers (ARB)] and kidney length was measured by ultrasound (Westland et al., 2011, 2013a). The KIMONO study showed that approximately one in three children with a SFK had signs of kidney injury at a mean age of 6.4 years (Westland et al., 2013a). Moreover, 26% of children with a SFK developed hypertension as early as 5 years of age, and 19% had proteinuria by 10 years of age (Westland et al., 2013a). In a small portion of children (6%) GFR had begun to decline at a mean age of 6.4 years (Westland et al., 2013a). Kaplan–Meier analysis showed that the median age to develop kidney injury in children with a SFK was ∼15 years (Westland et al., 2013a). Furthermore, children with ipsilateral CAKUT (34% of all children with SFK) had a significantly higher incidence of kidney injury and a younger median age to develop kidney injury (12.8 years) compared with children without ipsilateral CAKUT (Westland et al., 2013a). In the KIMONO cohort a higher proportion of children with a SFK were boys (65%), but no clear influence of gender on the development of kidney injury in SFK was found (Westland et al., 2013a; Westland and Schreuder, 2014).

In other studies the prognosis for children with a congenital SFK is suggested to be more favorable. Indeed, in a recent retrospective study of ∼300 children prenatally diagnosed with a congenital SFK, only 3.9% had signs of kidney injury by a median age of 7 years (Marzuillo et al., 2017). Similarly, La Scola et al. (2016) observed that in a cohort of 146 children with a congenital SFK, only 5% of patients had hypertension at a mean age of 12 years and only 4% presented with proteinuria at a mean age of 6 years. However, a more recent study by La Scola et al. (2020) found that 33% of SFK patients were hypertensive. This was based on ambulatory blood pressure measurement, which did show many cases of masked hypertension, similar to the KIMONO study (Westland et al., 2014; La Scola et al., 2020). The inconsistencies in cardiovascular and renal outcomes observed between studies may therefore reflect differences in study parameters such as inclusion criteria, selection bias, age of follow-up, and methods used to determine blood pressure, GFR and proteinuria. Despite these discrepancies, collectively in these studies it was shown that a congenital SFK does incur a greater predisposition for hypertension and kidney dysfunction later in life. However, there is a need for large long-term follow up studies beyond adolescence in individuals with a congenital SFK, as kidney dysfunction may develop later in life (Kolvek et al., 2014; Schreuder, 2017). It would be highly desirable to determine at diagnosis of SFK, which children have a poor prognosis and for this, accurate and sensitive biomarkers need to be identified.

## Congenital SFK Versus Early Acquired SFK

Although data is limited, there appears to be differences in the renal outcomes associated with the origin of SFK. In the KIMONO cohort those individuals with an early-acquired SFK had a greater incidence of kidney injury compared with those with a congenital SFK (Westland et al., 2011, 2013a). Consistent with these data, Abou Jaoudé et al. (2011) reported an ∼11% lower GFR in children with an early-acquired SFK (*n* = 53) compared with a congenital SFK (*n* = 44). A caveat to the finding of the KIMONO study is that those with an early-acquired SFK were older at follow-up and had greater incidence of ipsilateral CAKUT compared with a congenital SFK (Westland et al., 2011, 2013a). As both older age and ipsilateral CAKUT were independent risk factors for kidney injury in the KIMONO cohort, these may account for the greater incidence of kidney injury in individuals with an early-acquired SFK.

## Preclinical Model Systems

Over the last 20 years our group has characterized the phenotype of a congenital SFK in sheep. This model is generated by removal of a kidney from the sheep fetus at gestation day 100, during the period of nephrogenesis in the sheep (term = 150 days). There are various advantages of using the sheep as a model of SFK; (1) the development of the kidney in sheep is similar to that in humans with nephrogenesis reaching completion prior to birth in both species; (2) the size of the sheep and blood pressure and kidney function parameters are similar to that in humans making it an important preclinical model to examine the alterations in mechanisms regulating glomerular and tubular function and how these may underlie kidney injury in children with SFK (Table 2) (Moritz et al., 2008). Male and female rat pups that underwent unilateral nephrectomy in the first 24 h of postnatal life, a time when nephrogenesis is ongoing, have elevated blood pressure and lower GFR at the age of 20–22 weeks when compared with controls. In these uninephrectomized males, the elevation in blood pressure was greater than counterpart females and proteinuria and glomerular pathology was only observed in the males (Woods, 1999; Woods et al., 2001b). Similarly, in adult rats compensatory kidney hypertrophy of the remaining kidney 8 weeks after unilateral nephrectomy was two times greater in males compared with females and glomerular hypertrophy and damage were only observed in males but not in females (Mulroney et al., 1999). These observations suggest that SFK may increase disease severity in males more than females but these need to be confirmed in studies in humans.

**TABLE 2:** Comparison of the features in human and ovine fetal SFK.

Characteristics	Congenital SFK					
	Children	Age at onset	References	Ovine (males only)	Age at measurement	References
Elevated blood pressure	√	Mean 4.9 years	Westland et al. (2013a)	√ (7–15 mmHg)	6 months	Singh et al. (2010)
Reduced GFR	√	Mean 6.4 years	Westland et al. (2013a)	√ (30% decrease)	6 months	Singh et al. (2010)
Albuminuria	√	Mean 9.8 years	Westland et al. (2013a)	√	6 months	Singh et al. (2009)
Kidney hypertrophy	√	20–36 weeks gestation	Van Vuuren et al. (2012b)	√	130 days gestation	Douglas-Denton et al. (2002)

*GFR, glomerular filtration rate.*

## Adaptations to a SFK

It is well established that following a reduction in kidney mass whether it is congenital in origin or following unilateral nephrectomy in the child or the adult, there is marked compensatory kidney growth that is comprised of hypertrophy of both the kidney tubules and the glomerulus (Moritz et al., 2002, 2005; Singh et al., 2010; Fong et al., 2014; Chen et al., 2016; Wang et al., 2019; Zambaiti et al., 2019). In humans, compensatory kidney hypertrophy of the SFK begins as early as 20 weeks into gestation and by 36 weeks into gestation there is an ∼11% mean enlargement of the SFK compared with age matched controls (Van Vuuren et al., 2012b). Compensatory nephrogenesis is a characteristic adaptation to loss of kidney mass *in utero* but this is not always accompanied with compensatory increases in size of glomeruli. In a single human case study of a congenital SFK autopsied from a healthy 27-year-old male, it was observed that the congenital SFK weighed twice as much and had twice as many nephrons as a single kidney from an age-matched control (Maluf, 1997). In a study in 26-week-old pigs born with a congenital SFK, an 80% increase in kidney weight and using stereology, a 50% increase in nephron number in the SFK compared with a single control kidney was observed but individual glomerular volumes were similar to that of control (Van Vuuren et al., 2012a). In our ovine model of congenital SFK, fetal unilateral nephrectomy at 100 days of a 150-day gestation, resulted in a ∼45% greater nephron number in the SFK compared with a single kidney of a sham operated control sheep at 130 days of gestation but individual glomerular volumes were lower in the SFK compared with control at this age (Douglas-Denton et al., 2002). In contrast, unilateral nephrectomy after birth has been shown to result in increase in glomerular size in the rat and the mouse (MacKay et al., 1990; Nyengaard, 1993; Mulroney et al., 1999; Woods, 1999; Woods et al., 2001b).

In addition to the compensatory increase in size of glomeruli, an increase in single nephron filtration (glomerular hyperfiltration), and increases in size and function of kidney tubules are also characteristic responses to loss of kidney mass (Layton et al., 2017). These include increases in the diameter and length of the proximal and distal tubules and density of sodium transporters, all of which facilitate a greater reabsorption of the increased filtered load (Celsi et al., 1986; Shirley and Walter, 1991; Pollock et al., 1992) (see Table 3). These have been reviewed in detail elsewhere (Fong et al., 2014; Lankadeva et al., 2014) and will be discussed briefly here. The increase in filtration and increase in function of the kidney tubules facilitates compensation of total GFR such that GFR of a single kidney is relatively the same as that of two kidneys. In landmark studies, originally in 5/6th nephrectomized rats, Brenner hypothesized that a reduction in kidney mass and thus nephron number result in adaptive increases in glomerular capillary pressure and glomerular hypertrophy facilitated glomerular hyperfiltration (increases in single nephron GFR; SNGFR), thus maintaining GFR within normal levels (Brenner et al., 1988). The age at which reduction in kidney mass occurs may strongly influence these compensatory responses with evidence that the compensation is more robust when kidney mass is reduced in the young compared with the adult (Larsson et al., 1980). Indeed, following donation of a kidney in adult humans, GFR recovers to ∼70% of pre-donation values (Krohn et al., 1966; Fesler et al., 2015). In contrast, in children (∼9 years old) with a congenital or early-acquired SFK, GFR is preserved at a normal two-kidney level (100% increase in GFR) (Abou Jaoudé et al., 2011). These observations are also supported by various studies in animals in which GFR and kidney size have been shown to increase more in the young after loss of a kidney compared with the adult (Galla et al., 1974; Larsson et al., 1980; Shirley and Walter, 1991) (see Table 3).

**TABLE 3:** Summary of compensatory adaptations to reduction in kidney mass.

Species	Variable	Age at nephrectomy	Age at measurement	Outcomes	References
Rat	Kidney weight	5 days 12 days 40 days	60 days old	72% increase 65% increase 36% increase	Larsson et al. (1980)
		Weanling Young adult	4 weeks post-unilateral nephrectomy	144% increase 66% increase	Galla et al. (1974)
Rat	Glomerular volume	3 days 120 days	540 days	59% increase 20% increase	Nyengaard (1993)
Human	Total GFR	Congenital 20–60 years	9 years old 1–18 days post-unilateral nephrectomy	100% increase 70% increase	Abou Jaoudé et al. (2011)Krohn et al. (1966)
Rat	SNGFR	5 days 12 days 40 days	60 days old	115% increase 74% increase 47% increase	Larsson et al. (1980)
Rat	Proximal tubule length	Adult	2–4 weeks post-unilateral nephrectomy	35% increase	Hayslett et al. (1968)
		Adult	4–6 post-unilateral nephrectomy	71% increase	Pollock et al. (1992)
		5 days old	8 weeks	160%	Celsi et al. (1986)
Rat	Sodium reabsorption	Adult	4–6 weeks post-unilateral nephrectomy	50% increase	Pollock et al. (1992)
	Fractional proximal reabsorption	Adult	30 days post-unilateral nephrectomy	40% increase	Shirley and Walter (1991)
Rat	Sodium transporter density	5 days old	2 weeks post-unilateral nephrectomy	168% increase	Celsi et al. (1986)

*GFR, glomerular filtration rate; SNGFR, single nephron glomerular filtration rate.*

## Mechanisms Facilitating Adaptations and Potentiating Kidney Dysfunction

Increases in SNGFR facilitate adaptations compensating for loss of functioning nephrons but may also drive glomerular injury and contribute to progressive loss of kidney function (Brenner et al., 1988). Direct measurements of SNGFR are not possible in humans. Total GFR (the sum of all SNGFR) is currently the best prognostic indicator of kidney function in humans (Denic et al., 2017). SNGFR is influenced by various intrarenal mechanisms including glomerular capillary pressure, single nephron plasma flow, and tubuloglomerular feedback (TGF) (Blantz and Pelayo, 1984). The rise in SNGFR and glomerular capillary pressure following reduction in kidney mass has been associated with a reduction in afferent arteriole resistance and a right-ward shift in TGF (Müller-Suur et al., 1980; Salmond and Seney, 1991; Brown et al., 2011; Monu et al., 2018). It has been suggested that the right-ward shift results in blunting (resetting) of TGF where significantly higher fluid flow rates are required to cause a TGF mediated inhibition of SNGFR (Salmond and Seney, 1991). Thus, this resetting of TGF permits the increase in SNGFR and increase in glomerular capillary pressure in the remaining nephrons following nephron loss. In the rat, resetting of TGF 24 h after unilateral nephrectomy is mediated by enhanced connecting tubule glomerular feedback (a feedback system that influences afferent arteriole dilatation and is associated with epithelial sodium transporter; ENaC) (Monu et al., 2018). This resetting of TGF also sustains the increase in glomerular capillary pressure observed following 5/6th and unilateral nephrectomy in the rat (Salmond and Seney, 1991). Since increases in glomerular capillary pressure can increase injury to the glomeruli it can be suggested that resetting of TGF may contribute to glomerular injury following loss of nephrons (Hostetter et al., 1981).

The age-related differences in glomerular hyperfiltration following a reduction in kidney mass may also be associated with differences in the glomerular hemodynamic responses driving glomerular hyperfiltration. In an adult rat model of 5/6th nephrectomy with a two and a half fold increase in SNGFR compared with controls, glomerular capillary pressure was only increased by 10% (Bidani et al., 1990). In contrast, guinea pigs subjected to unilateral nephrectomy at birth had a 30% increase in glomerular capillary pressure at 10–21 days of age (Chevalier, 1983). Interestingly, Celsi et al. (1989) observed that in 20-day-old rats, subjected to unilateral nephrectomy at 5 days of age, the two-fold increase in SNGFR was maintained by an increase in glomerular ultrafiltration pressure. However, by 60 days of age, this increase in SNGFR was maintained by both an increase in glomerular ultrafiltration pressure and increase in the filtering surface area (Celsi et al., 1989). These data suggest that a nephron loss in the young may favor a compensatory increase in glomerular capillary pressure to maintain GFR in the short-term (Celsi et al., 1989). In contrast, in a long-term study of glomerular hemodynamics in adult kidney donors, Lenihan et al. (2015) inferred that following kidney donation compensatory glomerular hyperfiltration was maintained predominantly by glomerular hypertrophy rather than an increase in glomerular capillary pressure. In a Japanese cohort consisting of normotensives, hypertensives and subjects with CKD, Kanzaki et al. (2017) reported that individuals with a history of CKD had half as many nephrons per kidney (55%) as age matched normotensive individuals and 30% fewer nephrons per kidney than hypertensive individuals. In hypertensive subjects the volume of individual non-sclerosed glomeruli were greater than that of normotensive subjects but volume of non-sclerosed glomeruli was greatest in subjects with CKD. Estimated SNGFR was similar between subjects with hypertension and CKD but greater than that of normotensive subjects (Kanzaki et al., 2017). This suggested that increases in glomerular size supported GFR in the presence of low nephron number. Compensatory adaptations in size and function of kidney tubules facilitate a greater reabsorption of the increased filtered load (Celsi et al., 1986; Shirley and Walter, 1991; Pollock et al., 1992) (see Table 3). An increase in sodium transporter expression has been reported following reduction in kidney mass (Amaral et al., 2009; Kim et al., 2010; Singh et al., 2010). In our ovine model of congenital SFK, associated with elevated blood pressure and reduced GFR we have shown increased expression of Na+/K+ATPase β1, Na+/K+ATPase γ subunits and type 3 sodium hydrogen exchanger (NHE3) at 6 months of age (Singh et al., 2010). Moreover, following subtotal nephrectomy in the rat, at 4 weeks of age elevation in expression of Na-K-2Cl cotransporter and Na-Cl cotransporter was observed but these declined in expression by 12 weeks of age and this change in expression was associated with elevation in sodium excretion and increased kidney damage (Kim et al., 2010). This suggests there may be an association between changes in renal sodium transporter expression and severity of kidney injury. A greater reabsorption of certain components of the filtered load may also predispose the tubules to injury. For example, excessive proximal tubular reabsorption of filtered proteins or protein-bound substances in disease states with proteinuria has been shown to cause accumulation of inflammatory cells and macrophages in the tubular interstitium which may in turn enhance synthesis of extracellular matrix and proliferation of interstitial fibroblasts resulting in tubular injury (Eddy, 1994; Kees-Folts et al., 1994; Benigni and Remuzzi, 1996).

The underlying stimulus and mechanisms mediating kidney hypertrophy remain unclear. In the pig, compensatory nephrogenesis resulting in 50% increase in nephron number was associated with 1.4-fold increase in the number of medullary papillae (Van Vuuren et al., 2012a). Using ultrasound, a similar 1.3-fold increase in number of medullary papillae has also been observed in human fetuses with a congenital SFK (24 weeks gestation) compared with control fetuses (Snoek et al., 2018). Increase in number of medullary papillae indicates an increase in ureteric bud arborization, a process that determines final nephron complement suggesting that enhanced ureteric branching morphogenesis facilitates compensatory nephrogenesis in congenital SFK. These findings also suggest that the total nephron endowment in congenital SFK will amount to ∼70–75% rather than 50% of full nephron complement.

Other factors such as the nitric oxide system, the renal sympathetic nerves and the renin angiotensin system (RAS) have been implicated in the compensatory adaptations to reduction in kidney mass (Rojas-Canales et al., 2019). Nitric oxide (NO), released by the endothelium, is an important vasodilator of the vasculature. NO and the by-product L-citrulline, is formed from the precursor L-arginine by NO synthase (NOS), of which there are three isoforms, neuronal NOS (nNOS), inducible NOS (iNOS), and endothelial (eNOS) (Mount and Power, 2006). The expression of all three NOS isoforms have been observed in the kidney, and as such NO plays an important role in the modulation of renal glomerular hemodynamics, tubular function, fluid composition and volume and thus arterial pressure (Denton and Anderson, 1994; Mount and Power, 2006). The kidney hypertrophic response following unilateral nephrectomy in the rat has been suggested to be mediated by an elevation in production of NO and increase in renal blood flow (RBF) (Sigmon et al., 2004). The increased RBF following unilateral nephrectomy has also been proposed to increase the delivery of free amino acids to the remaining kidney stimulating the mTORC1 pathway which induces protein synthesis and cell proliferation leading to kidney hypertrophy (Chen et al., 2015). Blockade of NO with L-NAME prevents this increase in RBF and kidney hypertrophy (Sigmon et al., 2004). In endothelial nitric oxide (eNOS) knockout mice, an increase in kidney weight and increase in RBF were not observed after unilateral nephrectomy whereas in eNOS transgenic mice with targeted overexpression of eNOS to the vascular endothelium, an increase in compensatory kidney growth was observed after unilateral nephrectomy compared with the sham procedure (Nagasu et al., 2012). These collectively suggest that eNOS facilitated increase in RBF and enhanced expression of eNOS promotes compensatory kidney hypertrophy. Conversely, a reduction in NO has also been observed in animal models of reduced kidney mass and in humans with kidney failure and CKD (Blum et al., 1998; Schmidt and Baylis, 2000). At 15 weeks after 5/6th nephrectomy in rats, accompanying decreases in creatinine clearance, increases in proteinuria and glomerulosclerosis, total NO production and renal cortical nNOS were reduced (Tain et al., 2007). In our ovine model of SFK, we have demonstrated a reduced contribution of NO to kidney function in both aged and young sheep (Lankadeva et al., 2015; Singh et al., 2016). In sheep with congenital SFK, the renal responses to systemic blockade of NOS, via the administration of L-NAME, were attenuated compared with sham-operated sheep despite increased expression of renal eNOS (Lankadeva et al., 2015). The increase in expression of eNOS suggested that the kidneys could produce NO but further studies identified that increased oxidative stress maybe contributing to the reduction in bioavailability of NO and reducing the contribution of NO to kidney function in the sheep with SFK (Lankadeva et al., 2015). In addition to the reduced contribution of NO to kidney function, we have demonstrated reduced total urinary nitrate/nitrite levels in sheep with SFK (Singh et al., 2016). From these collective observations, we speculate that increases in eNOS may promote increases in RBF and promote the renal hypertrophic adaptation to SFK in the short-term but that reduction in bioavailability of NO, probably associated with increased oxidative stress may contribute to kidney dysfunction and hypertension in SFK.

Indirect evidence has also implicated renal sympathetic nerve activity (RSNA) in the kidney hypertrophic response (Furukawa et al., 1997). Following unilateral nephrectomy in the rat, relative mean RSNA increased by 78% at 28 days after nephrectomy, and this was associated with a 62% increase in kidney weight (Furukawa et al., 1997). Although we do not have direct evidence that elevations in RSNA drive compensatory hypertrophy, we do have evidence of renal hyperinnervation in our ovine model of SFK (Singh et al., 2017, 2019). In our recent studies, we have demonstrated sheep with a congenital SFK have a greater proportion of both sympathetic renal nerves and sensory renal nerves than sham animals demonstrating renal nerve hyperinnervation in SFK (Singh et al., 2019). The spontaneously hypertensive rat (SHR) model is a genetic model of hypertension and there is evidence that the kidneys of the SHR have less nephrons than normotensive Wistar Kyoto (WKY) control counterparts (Skov et al., 1994). In this model, accelerated development of renal innervation has been observed from newborn to postnatal week 6 compared with WKY controls and sympathetic innervation induced changes in kidney function in the neonate contribute to development of hypertension in SHR (Gattone et al., 1990; Grisk et al., 2002). Evidence for contribution of overactivity of the renal nerves to hypertension and kidney failure has also been documented in humans (Converse et al., 1992; Grassi et al., 2011; Symplicity, 2011; Krum et al., 2014; Mauriello et al., 2015). Collectively these findings suggest that renal hyperinnervation may have a role in promoting renal and cardiovascular dysfunction in models of reduced nephron number and as such have implications for SFK.

The RAS is a potent modulator of blood pressure, glomerular hemodynamics and fluid and electrolyte homeostasis and is also an important modulator of kidney development and maturation in the fetus and the neonate (Chen et al., 2004). Considerable evidence has also established the role of the RAS in the pathophysiology of hypertension and kidney disease (Hall et al., 1978; Johnson et al., 1992; Woods et al., 2001a; Grigore et al., 2007; Cuffe et al., 2016; Walton et al., 2017). Our ovine model of SFK is a model of low-renin hypertension, where we have reported lower levels of plasma renin in sheep with SFK from 6 months of age in both males and females (Moritz et al., 2002; Singh et al., 2009). This reduction in renin is likely a normal response to the rise in blood pressure that is observed in the sheep with SFK from 6 months of age (Singh et al., 2009). Additionally, we have also demonstrated that regulation of glomerular hemodynamics by the RAS is impaired in sheep with SFK, suggesting that this may potentially be a system that can be targeted to improve renal outcomes in SFK (Singh et al., 2011). Postnatal adaptive maturation of both blood pressure and glomerular filtration rate (GFR) is associated with the RAS (Robillard et al., 1983; Prevot et al., 2002; Vinturache and Smith, 2016). In conscious lambs, blood pressure is low at birth and plasma renin activity is elevated but during the first 3 months of post-natal maturation, plasma renin activity decreases as blood pressure increases (Monument and Smith, 2003; Velaphi et al., 2007). Additionally, in the early postnatal period in conscious lambs angiotensin II activation of AT1R regulates GFR and kidney injury associated with glomerular hyperfiltration and hypertrophy may be underpinned by RAS activation (Babić et al., 2007; Vinturache and Smith, 2016).

## Biomarkers and Areas of Future Study

Classic biomarkers of CKD (albuminuria, reduced eGFR) occur at advanced stages of disease progression. Therefore, advancements in genetic testing, proteomics, metabolomics and imaging techniques may offer prognostic markers to predict CKD risk and disease progression before decline in kidney function occurs, allowing for the possibility of earlier intervention in SFK (Table 4) (Greenberg et al., 2018; Bennett et al., 2020).

**TABLE 4:** Current and next generation prognostic biomarkers for progression of kidney injury in children with SFK.

Biomarker	Access	Cost	Prognostic utility	References
**Kidney mass and kidney length** Ultrasound	Freely accessible	Inexpensive	Children with a smaller SFK are at a greater risk for kidney injury	Wang et al. (2010); Westland et al. (2013a)
**Nephron number** MRI	Under development- research testing in animals only	Expensive	May assist in differentiating those with a reduced nephron number early in life	Beeman et al. (2014); Baldelomar et al. (2018)
**Hyperfiltration** eGFR SNGFR Urinary prostaglandin E2 Urinary nitrates	Freely accessible	Inexpensive	Early changes in markers could predict risk of kidney disease	Westland et al. (2013a); Singh et al. (2016); Srivastava et al. (2020)
**Plasma** Creatinine Cystatin C KIM-1 MCP-1	Freely accessible	Inexpensive	Kidney dysfunction and tubular injury	Wasilewska et al. (2006); Greenberg et al. (2020)
**Urine** Creatinine eGFR Albuminuria *N*-Acetyl-beta-D-glucosaminidase (NAG) *N*-Acetyl-β-hexosaminidase (HEX)	Freely accessible	Inexpensive	Glomerular and tubular injury Predict risk of kidney injury	Gadalean et al. (2013); Westland et al. (2013a); Taranta-Janusz et al. (2014)

*MRI, magnetic resonance imaging; GFR, glomerular filtration rate; eGFR, estimated glomerular filtration rate; SNGFR, single nephron glomerular filtration rate; KIM-1, kidney injury molecular-1; MCP-1, monocyte chemoattractant protein-1.*

### Nephron Number

A deficiency of nephrons is a risk factor for kidney disease but the threshold of nephron number that predisposes to kidney disease is currently not known (Bertram et al., 2011). Harnessing this knowledge has been limited to-date given that nephron number cannot be determined *in vivo* in living subjects. Magnetic resonance imaging (MRI) in combination with cationized ferritin labeling of glomeruli using both *ex vivo* and *in vivo* approaches has been established as a reliable determinant of nephron number in rodents (Geraci et al., 2017; Baldelomar et al., 2018). Similarly, MRI in combination with cationized ferritin has been utilized to determine nephron number *ex vivo* in the whole kidney of the human (Beeman et al., 2014). Continued progress in this field resulting in determination of nephron number in the living human kidney will improve our ability to identify individuals at risk of developing kidney disease and track the progression of disease. This approach may also assist us in differentiating the long-term cardiovascular and renal outcomes between early-acquired SFK and a congenital SFK. It is known that the kidney of the human does not have the capacity to form new nephrons after birth (Moritz and Wintour, 1999). Thus, the degree of nephron deficit may be more severe in an early-acquired SFK than a congenital SFK, and this may potentially underpin the greater risk for kidney injury in the early-acquired SFK group (Westland et al., 2014; Schreuder, 2017). The ability to determine a threshold for nephron number that predisposes to disease will enable is to devise better treatment strategies in individuals with different origins of SFK.

### Kidney Size

In the absence of direct measurements of nephron number and limited information on validity of biomarkers for predicting kidney injury/disease in SFK, kidney size remains an important predictor of kidney injury in SFK. Inadequate kidney growth indicative of reduced nephron hyperplasia and/or hypertrophy has become an important predictive index of kidney dysfunction in children with a SFK (Westland et al., 2013a; Marzuillo et al., 2019; Poggiali et al., 2019). In a small cohort of Chinese adults with a congenital SFK, it was observed that individuals with a kidney length less than 120 mm had a five-fold greater risk for impaired GFR (<60 ml/min/1.73 m^2^) (Wang et al., 2010). In the KIMONO cohort, it was demonstrated that a smaller SFK compared with a larger SFK was associated with a greater risk of developing kidney injury (Westland et al., 2013a). Similarly, La Scola et al. (2016) observed that a kidney length less than 10% expected for a congenital SFK was the strongest predictor of reduced eGFR by 10 years of age. Conversely, Marzuillo et al. (2019) found that children who were diagnosed prenatally with SFK and had a greater than two standard deviation score (+2-SDS) kidney length in the neonatal period (within 60 days of life) were at a reduced risk of having lower eGFR at the age of 15 years compared with those with a kidney length less than + 2-SDS. Importantly, in this study, it was demonstrated that the risk for low eGFR in children with a kidney length < 2-SDS presenting with postnatal compensatory kidney hypertrophy was similar to those with kidney length < 2-SDS but without postnatal compensatory kidney hypertrophy. Based on these findings the authors concluded that nephron hyperplasia resulting in a greater kidney length likely provided protection against loss of kidney function in the long-term but nephron hypertrophy did not offer this protection (Marzuillo et al., 2019). The importance of kidney length as a predictor of kidney function in SFK has been further highlighted by a study by Wang et al. (2019) who demonstrated that every 1 cm increase in kidney length predicted a 7.8 ml/min/1.73 m^2^ increase in GFR in children with a SFK. Together these studies suggest that insufficient compensatory kidney growth; perhaps lack of nephron hyperplasia more so than nephron hypertrophy, may increase the susceptibility to kidney dysfunction. However, it has been found that kidney size estimations in adults (with two kidneys) has poor predictive value in estimating nephron number, with only 5% of the variation in nephron number explained by the variation in kidney size (Bueters et al., 2013). This again highlights the need for determining nephron number together with kidney size in the living human, as these may be important predictive markers that can be utilized to identify those at an elevated risk for future kidney injury.

### Glomerular Injury

**FIGURE 1:**
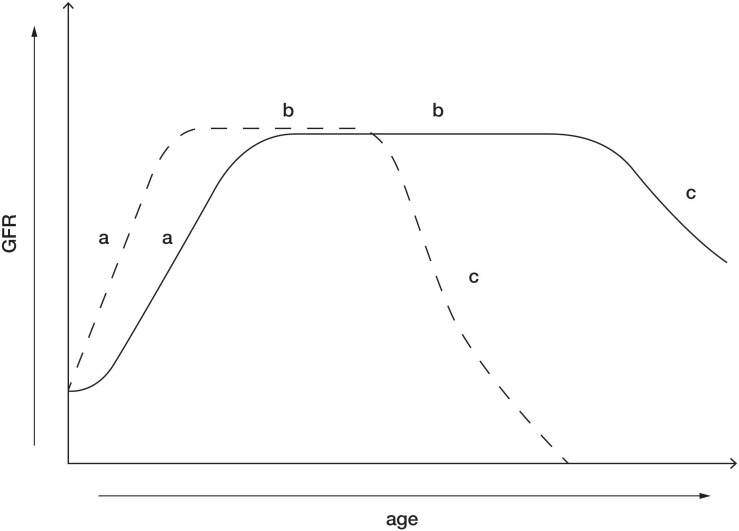
Postnatal maturation of kidney function in healthy individuals with two kidneys (solid line) and maturation of kidney function in children with a congenital SFK (dotted line). (a) The phase after birth when GFR increases. This phase is likely enhanced in children who lose a kidney early in life. (b) GFR reaches its peak and is maintained within normal range for most of life in the absence of a “second hit” such as obesity, hypertension, and diabetes. This phase is likely to be shorter in children with SFK, possibly due to enhanced compensatory adaptations increasing the risk of kidney dysfunction. (c) Age-related decline in GFR in kidneys. This phase may be accelerated in individuals with SFK with kidney dysfunction and in some cases may result in kidney failure.

Mechanisms pertaining to glomerulosclerosis associated with reduction in kidney mass are under investigation and implicate a role for podocyte loss in glomerular injury and dysfunction. During glomerular hypertrophy/hyperfiltration, associated increases in glomerular capillary pressure, exposes podocytes to stretch, tensile stress and fluid flow shear stress (FFSS), which may overtime decrease the integrity of the glomerular filtration barrier, resulting in podocyte damage and loss (Nagata et al., 1992; Srivastava et al., 2014b, 2017; Mallipattu and He, 2016). Following unilateral nephrectomy in young rats it has been observed that increases in SNGFR mediate a 1.5- to 2-fold increase in FFSS over podocytes and isolated glomeruli exposed to FFSS for 2 h have increased permeability to albumin indicating damage to the glomerular filtration barrier (Srivastava et al., 2014a, b). Additionally, in *in vitro* studies, applying FFSS to cultured podocytes has been demonstrated to increase levels of prostaglandins (PGE_2_) from as early as 30 min after induction of FFSS and has been demonstrated to cause a reduction in the actin cytoskeleton of the podocytes (Srivastava et al., 2010). This suggests that PGE_2_ levels may serve as a marker of hyperfiltration mediated FFSS and glomerular filtration barrier dysfunction. In a recent study in children with SFK, it was shown that urinary PGE_2_ and albumin levels were elevated in children with a SFK (*n* = 57), at a mean age of 8.5 years, compared with children with two kidneys but it was also demonstrated that albuminuria was preceded by elevations in urinary PGE_2_ (Srivastava et al., 2020). Therefore, elevated urinary PGE_2_ may be a potential biomarker for FFSS associated with hyperfiltration and may predict risk of kidney injury in children with a SFK (Srivastava et al., 2020). Moreover, since the increase in SNGFR after reduction in kidney mass are greater in the young compared with the adult (Table 2), these data suggest that adaptive glomerular hyperfiltration and associated increases in FFSS sustained from a younger age may cause degradation of the glomerular filtration barrier earlier in life, resulting in podocyte loss and may account for the greater incidence of kidney injury in children with a SFK compared with adult kidney donors (Figure 1).

### Tubular Injury

In the CKD in Children (CKiD) multicenter cohort center, plasma concentrations of proximal tubular injury (kidney injury molecular-1, KIM-1), pro-inflammatory markers (monocyte chemoattractant protein-1, MCP-1) and receptors for proinflammatory markers (tumor necrosis factor alpha; TNFR1 and TNFR2) in the highest quartile identified children at greater risk of CKD progression compared with those with concentrations in the lowest quartile (Greenberg et al., 2020). Gadalean et al. (2013) observed that in adult patients with an acquired and congenital SFK there was a ∼52–60% increase in urinary *N*-acetyl-beta-D-glucosaminidase, a marker of proximal tubule dysfunction. Similarly, in children with both a congenital SFK and acquired SFK, urinary *N*-acetyl-β-hexosaminidase (HEX), an indicator of proximal tubular damage, was elevated compared with age-matched healthy individuals (Taranta-Janusz et al., 2014). Moreover, estimated urinary HEX was a good diagnostic indicator of kidney injury (presence of albuminuria) in children with a SFK (Taranta-Janusz et al., 2014). Interestingly, it has been observed that children with URA excrete higher levels of β2 -microglobulin indicating proximal tubular damage, compared with children with an early-acquired SFK (unilateral nephrectomy due to Wilm’s tumor) (Stefanowicz et al., 2012). These data indicate that a greater reabsorption of filtered load may increase tubulointerstitial inflammation resulting in tubular injury and that urinary markers of proximal tubular injury are elevated in children with a SFK and may be diagnostic indicators of kidney damage.

### Endothelial Dysfunction

Asymmetrical dimethylarginine (ADMA), a competitive NOS inhibitor that causes reduced NO production, and its enantiomer symmetric dimethylarginine (SDMA) have been observed to accumulate in the early stages of CKD and kidney failure (Zoccali et al., 2001; Kielstein et al., 2002; Fleck et al., 2003; Busch et al., 2006; Brooks et al., 2008; El-Sadek et al., 2016). Ravani et al. (2005) demonstrated that plasma ADMA is a strong independent risk factor for progression to kidney failure and death in patients with CKD. Brooks et al. (2008) observed reduced plasma arginine and elevated ADMA and SDMA in pediatric/adolescent CKD (stage 2–3) patients, with SDMA demonstrated to be a strong indicator of reduced GFR. Importantly, in a small cohort of 51 children with a SFK at a mean age of ∼10 years, plasma SDMA was significantly elevated and negatively correlated with GFR, suggesting that plasma SDMA may serve as a marker of endothelial dysfunction in children with a SFK (Taranta-Janusz et al., 2012).

## Potential Treatment Options

### Increasing NO Bioavailability

Nitric oxide has an important role in the renal hypertrophic and hemodynamic adaptation in SFK. However, reductions in bioavailability of NO may be associated with increases in oxidative stress in SFK (Lankadeva et al., 2015). Increasing bioavailability of NO by dietary supplementation with L-arginine in rats subjected to subtotal nephrectomy has been shown to prevent glomerular hypertension and preserve kidney function and has been shown to prevent glomerular hyperfiltration and proteinuria in diabetic rats (Reyes et al., 1993; Katoh et al., 1994). In hypertensive patients with micro-vascular angina, oral L-arginine treatment for 4 weeks was shown to improve systolic blood pressure and quality of life (Palloshi et al., 2004). The therapeutic value of oral-L-arginine treatment needs to be further explored in SFK.

### Renal Denervation

In our recent studies, we have demonstrated that renal denervation in sheep with SFK resulted in long-term lowering of blood pressure, improvement of GFR and reduction in albuminuria compared with animals with SFK that underwent a sham denervation procedure (Singh et al., 2019). This suggests that hyperinnervation of the renal nerves may potentially be contributing to the elevation in blood pressure and kidney disease in SFK and has implications for children with SFK. A role for renal sympathetic overactivity in promoting hypertension is also evident in other models of reduced kidney mass, such as the developmental programming models (maternal glucocorticoid exposure) and genetic models (SHR) (Gattone et al., 1990; Alexander et al., 2005; Dagan et al., 2008; Kett and Denton, 2011; Mansuri et al., 2017). Prenatal exposure to dexamethasone is well known to cause a reduction in nephron number and elevation in blood pressure in studies in various species (Singh et al., 2012). In the rat, the elevation in blood pressure in the offspring that results from prenatal exposure to dexamethasone was ameliorated by renal denervation (Dagan et al., 2008). Moreover, renal denervation in SHR reduces blood pressure long-term and attenuates the kidney injury associated with proteinuria and glomerulosclerosis (Wang et al., 2018). Combined with clinical evidence of the association of CKD with sympathetic hyperinnervation, markers of sympathetic activity in young children may serve as a prognostic indicator of future disease severity in children with a SFK. Indeed, noradrenaline can be detected in urine, the levels of which have previously been shown to correlate with the level of sympathetic activity (Lappe et al., 1982). Further studies examining the role of RSNA in models of reduced kidney mass are warranted in the future and may provide evidence to advance this to clinical studies.

### RAS Blockade

Pharmacological inhibition of the RAS with either an ACEi or an ARB may be effective in preventing hyperfiltration-mediated injury associated with SFK (Lewis et al., 1993; Romero et al., 2015). Indeed, in patients with diabetic nephropathy, in which hyperfiltration is a hallmark response, ACE inhibition prevents the decline in kidney function independent of its antihypertensive effects (Lewis et al., 1993). There is growing evidence that pharmacologically targeting the RAS during early postnatal maturation may provide long-lasting cardiovascular and renal benefits. Brief and early treatment with ACEi from 6 to 10 weeks of age in the SHR reduced blood pressure by ∼20–30 mm Hg until approximately 82 weeks of age (Harrap et al., 1994). Consistently, in models of maternal low protein diet or calorie restriction, treatment with ACEi or ARB’s for a brief period early in postnatal life prevented hypertension into early adulthood (Sherman and Langley-Evans, 1998, 2000; Manning and Vehaskari, 2005; Hsu et al., 2015). Interestingly, Hsu et al. (2015) observed that rat offspring exposed to maternal calorie restriction but treated with aliskiren, a renin inhibitor, from 2 to 4 weeks of age did not develop hypertension at 12 weeks of age. These improvements were associated with a reduction in angiotensinogen expression and improved bioavailability of NO. This suggests that the balance between NO bioavailability and RAS activity may influence regulation of blood pressure. Blockade of RAS in children with CKD and hypertension not only improves control of blood pressure but improves renal outcomes as well. Indeed, in the ESCAPE trial in children with CKD and hypertension, who received a high dose of an ACEi, blood pressure control was intensified and was associated with a 35% reduction in relative risk of loss of kidney function (50%) or kidney failure within the 5-year follow-up (Wuhl et al., 2009). In addition, a higher degree of early anti-proteinuric effects of ACEi predicted a lower risk of renal functional decline in these children with CKD (Van Den Belt et al., 2018). However, following 3 years of ongoing ACEi the proteinuria rebounded in the ESCAPE trial (Wuhl et al., 2009). The improvements in kidney outcomes associated with RAS blockade maybe associated with RAS control of glomerular hemodynamics. Anderson et al. (1985) observed that treatment with ACEi in a rat model of 5/6th nephrectomy controlled systemic and glomerular capillary pressure via efferent arteriole dilation which in turn prevented proteinuria and glomerular injury. Together this evidence indicates that RAS inhibition may prevent the rise in blood pressure and also alter glomerular hemodynamics in a manner that can reduce kidney injury in children with SFK.

## Conclusion

Children born with a SFK are at a greater risk for developing hypertension, kidney injury and CKD. This predisposition is likely associated with the greater degree of compensatory adaptations early in life that include compensatory increases in GFR, glomerular size and function and size of the kidney tubules following a reduction in kidney mass in young children or in fetuses. Our extensive studies in an ovine model of congenital SFK, along with work in other models of a reduction in kidney mass have demonstrated that alterations in the renal sympathetic nerves, the renal RAS and the nitric oxide system may be strong contributing factors to the development of disease associated with SFK. These studies have provided the foundation suggesting that these systems can potentially be targeted in SFK to reduce the progression of disease. Currently, common prognostic indicators such as hypertension, proteinuria and reduced GFR are available to detect kidney injury/failure in children with a SFK, but these emerge at later stages of disease progression. Emerging indicators such as kidney length and urinary markers of tubular injury and glomerular hyperfiltration may be beneficial in identifying those at an elevated risk of kidney injury earlier in life. However, markers that distinguish those at an elevated risk of kidney injury as opposed to those with a lower risk, before overt kidney injury occur, are needed. Ideally, this would occur during the neonatal period when the SFK was identified.

## Author Contributions

All authors assisted with drafting the manuscript, searching the literature and proofreading the document. All authors contributed to the article and approved the submitted version.

## Conflict of Interest

The authors declare that the research was conducted in the absence of any commercial or financial relationships that could be construed as a potential conflict of interest.
